# Effectiveness of a Brief Engagement, Problem-Solving, and Triage Strategy for High School Students: Results of a Randomized Study

**DOI:** 10.1007/s11121-022-01463-4

**Published:** 2023-03-17

**Authors:** Eric J. Bruns, Kristine Lee, Chayna Davis, Michael D. Pullmann, Kristy Ludwig, Mark Sander, Cheryl Holm-Hansen, Sharon Hoover, Elizabeth M. McCauley

**Affiliations:** 1 University of Washington School of Medicine, Seattle, USA; 2 Midwest Center for School Mental Health, St. Paul, USA; 3 University of Maryland School of Medicine, Baltimore, USA

**Keywords:** School mental health, Evidence-based practice, Measurement-based care, Stepped care, Brief treatment strategies, Adolescents, Effectiveness

## Abstract

Schools offer an advantageous setting for the prevention, early identification, and treatment of mental health problems for youth. However, school mental health (SMH) services are typically not based on evidence for effectiveness, nor are they efficiently delivered, with SMH practitioners (SMHPs) able to only treat a small number of students in need. The current study evaluated the feasibility, acceptability, efficiency, and outcomes of a four-session assessment, engagement, problem-solving, and triage strategy for SMHPs that aimed to improve efficiency while being based on elements of evidence-based care. The study, conducted in 15 US school districts in three states, used stratified random assignment to assign 49 high schools and their participating SMHP(s) to either the Brief Intervention for School Clinicians (BRISC; *N* = 259 students) or services as usual (SAU; *N* = 198 students). SMHPs implemented BRISC elements with adequate to excellent fidelity and reported the strategy was feasible and well-aligned with presenting problems. Students assigned to BRISC reported significantly greater engagement in SMH at 2 months and completion of SMH treatment by 6 months. BRISC-assigned SMHPs reported significantly greater treatment completion after four sessions (53.4%) compared to SAU (15.4%). Students in the BRISC condition also reported significantly greater reduction in problem severity as evaluated by the Youth Top Problems Assessment. No differences were found for anxiety or depression symptoms or overall functioning. Results indicate that BRISC is a feasible early intervention and triage strategy that may aid in more efficient provision of SMH services with no compromise to SMH effectiveness.

**Supplementary Information:**

The online version contains supplementary material available at 10.1007/s11121-022-01463-4.

Since 2010, rates of depression, anxiety, and suicidality in children and adolescents have risen to historic highs (Ivey-Stephenson et al., 2020; Yard et al., 2021). However, fewer than half of children and adolescents in need of mental health support receive any treatment (Whitney & Peterson, 2019). Schools offer an ideal setting for the provision of accessible and timely mental health services (Kataoka et al., 2007). School-based providers have access to students of all levels of need and can provide preventive and early intervention services not available in outpatient settings. Not only are schools the most common setting for the provision of mental health services for youth (Duong et al., 2020), youth receiving services in schools are more likely to complete treatment (Nadeem et al., 2011).

## Improving Efficiency and Effectiveness of School Mental Health Care

School-based services are characterized by large caseloads and clinician time constraints; they also tend to be crisis-driven (Langley et al., 2010; Lyon et al., 2014) and focused on providing nondirective emotional support (Holmes et al., 2021; Lyon et al., 2011) rather than evidence-based practices (EBPs) delivered with fidelity (Fazel et al., 2014). While school-based services provide substantial opportunity for prevention and early intervention, the large number of students in need demands systematic strategies that promote both efficacy and efficiency.

Education frameworks taking a broader public health approach, such as Multi-Tiered Systems of Supports (MTSS; National Center on Response to Intervention, 2010; Stephan et al., 2015), provide guidance for the integration of mental health delivery into schools. MTSS organizes available interventions across three tiers, with data used to screen for need, track effectiveness of interventions used, and need for additional or more intensive services. However, only an estimated 20% of school systems in the USA use routine screening much less have comprehensive mental health care systems (Herman et al., 2021).

While there is substantial support for the effectiveness of “Tier 1” universal strategies such as school-wide social-emotional learning programs (Durlak et al., 2011), there are fewer research-based strategies at Tiers 2 (indicated support) and 3 (targeted/intensive supports) (Bruns et al., 2019; Fazel et al., 2014). Most EBPs have not been designed to fit the needs of the school setting or developed in partnership with end users such as school-based providers and teachers (Holmes et al., 2021). Thus, efforts to use EBPs in schools have been characterized by “incomplete implementation, restricted sustainability, and narrow spread” (Fazel et al., 2014, p. 382).

Examples of efforts to tailor EBPs for use in school settings include adaptations of solution-focused brief therapy (SFBT). Early SFBT studies showed promise as a practical and efficient approach for students with internalizing and externalizing problems (Kim & Franklin, 2009). A more recent study found no effects on student symptoms but a significant reduction in absenteeism and teacher reports of improved classroom performance (Wallace et al., 2020).

A second relevant example is provided by “modular” approaches to therapy that use “common elements” of EBP tailored to the needs of a specific student. “Show Me FIRST” is a brief (6 session) school-based modular approach for depressed and anxious youth that incorporates strategies such as relaxation training, cognitive restructuring, problem solving, and behavioral activation. A pilot trial found support for feasibility but no differences on mental health outcomes compared to school-based usual care. A review concluded that modular approaches are potentially useful to SMHPs, but that more research is needed to determine if school-based providers, given the demands of their settings, can deliver these approaches effectively (Kininger et al., 2018).

In sum, whole-school frameworks such as applications of MTSS hold substantial promise for improving school-wide student MH outcomes. Such frameworks, however, also require targeted and intensive individual- and group-based SMH strategies for identified students that align with MTSS principles such as being research-based, data-informed, and provided within a tiered framework. Few models have been developed and rigorously tested for delivering SMH services that are both based on evidence for effectiveness as well as responsive to the vast and growing demands on SMHPs.

## Developing and Testing an Efficient SMH Model for “Real World” Schools

Working with local school districts, the current research team developed a SMH approach that incorporates problem-focused assessment, problem-solving, and flexibly applied evidence-based elements (Bruns et al., 2019; Lyon et al., 2015). Rather than providing lengthy episodes of care to a small cadre of youth, we aimed to base the approach on stepped care strategies (Asarnow et al., 2015)—in which an initial intervention requiring less therapist time is followed by more intensive and specialized intervention, only if required—as well as measurement and feedback strategies to inform such decisions. The ultimate goal was to minimize resource use and maximize the number of students served, as well as promote the MTSS principles of being based on data and provided within a tiered framework.

The current paper describes an initial efficacy test of this school-based triage and brief intervention approach. The Brief Intervention for School Clinicians (BRISC) provides a first-line, “Tier 2” intervention that aims to use consistent assessment to inform the level and type of ongoing services needed (including “Tier 3” services as needed) in a prevention-oriented, multi-tiered delivery model. BRISC incorporates modular elements of effective care (Weisz & Bearman, 2020) with good fit to the school context, including routine progress monitoring (Borntrager & Lyon, 2015), a brief functional assessment, identification of and focus on “top” problems identified by the youth (Weisz et al., 2011), and an overarching, research-based problem-solving framework (D'Zurilla & Nezu, 2010).

The BRISC theory of change posits two paths to positive outcomes. First, that SMHPs who use explicit engagement strategies, e.g., student’s identification of treatment target and ongoing data collection to guide decision-making will promote service outcomes such as greater alliance, treatment engagement and retention, and more appropriate and rapid referrals to other servies as needed. Second, that teaching effective problem solving and a targeted set of flexibly applied evidence-based strategies will promote better problem-solving, improved coping skills, and resolution of student top problems in the short term as well as connections to services if/as needed.

The study sought to answer the following research questions:

1. What were SMHPs’ perceptions of the *appropriateness*, *feasibility*, and *acceptability* of BRISC as a strategy for use with high school students referred to or seeking mental health treatment?
2. Compared to SAU, what were the effects of BRISC on *service processes*, including student-reported therapeutic alliance and satisfaction with SMH services?
3. Compared to services as usual (SAU), what were the effects of BRISC on *mental health services received*, including receipt of SMH and other services over time, rate of SMH treatment completion, and post-SMH treatment pathways?
4. Compared to SAU, what were the effects of BRISC on *student-identified top problems?*
5. Compared to SAU, what were the effects of BRISC on *mental health symptoms* and *functioning*?

We hypothesized that BRISC would be feasible and acceptable to SMHPs, promote greater initial engagement in SMH services and more rapid completion of care, and be at least equally effective in improving symptoms and functioning as SAU.

## Methods

This study was pre-registered at ClinicalTrials.gov (protocol number 52229). Block randomization occurred at the level of the school, and participants completed assessments at baseline, 3, and 6-months follow-up for the primary outcome measures of interest. Academic outcomes were collected; however, the current paper focuses on mental health services and outcomes only.

### Participants

#### Schools

The study was conducted in 15 school districts in 3 states: Maryland (13 schools, 2 districts), Minnesota (16 schools, 8 districts), and Washington (20 schools, 5 districts). School enrollment ranged from 67 to 2851 (*M* = 1,434, *SD* = 696) and average attendance from 64.5% to 97.0% (*M* = 90.9%, *SD* = 6.3%). Enrolled students ranged from 9.4 to 98.8% non-White (*M* = 47.7%, *SD* = 23.5%), 0.03 to 89.6% eligible for lunch subsidies (*M* = 31.3%, *SD* = 26.3%), 6.7 to 72.5% passing standardized tests for reading (*M* = 42.3%, *SD* = 14.2%), and 3 to 62% for mathematics (*M* = 39.9%, *SD* = 16.4%).

#### School Mental Health Practitioners (SMHPs)

All SMHPs in participating schools were included. Licensed mental health clinicians (*N* = 53) from community mental health agencies provided SMH services in 41 schools, while school counselors (*N* = 10) provided SMH services to students in 9 schools. SMHP age ranged from 25 to 61 (mean = 35.9, *SD* = 8.94), and years of experience in their role ranged from 1 to 22 years (mean = 5.56, SD = 5.50); SMHPs were primarily White (82.5%) and female (88.9%) and employed by a mental health agency external to the school district (69.8%).

#### Youth Participants

All students seeking or referred to services from participating SMHPs in participating high schools were eligible. Students were referred to or requested SMH through standard procedures used by their school/district. Students were ineligible if they currently or in the past year received therapy or counseling. Students in an acute crisis situation were also excluded. Students had to speak English; however, consent forms were translated and administered in Spanish so that students with Spanish-only speaking parents/legal guardians could be included.

### Measures

#### Mental Health Services Received

The Service Assessment for Children and Adolescents (SACA; Stiffman et al., 2000) was used to assess all mental health services received as well as mental health services received in school, outpatient, and inpatient settings. The SACA is a reliable and valid structured caregiver and youth interview; it asks 27 yes/no questions about whether the youth received a service (e.g., “Drug or alcohol clinic”). The SACA has been shown to have good concordance (*k* = 0.76) between caregiver reports and documentation of services (Horwitz et al., 2001).

#### Service Processes and Satisfaction

The Multidimensional Adolescent Satisfaction Scale (MASS) is a 21-item measure of client satisfaction with mental health services. Response options are on a 4-point scale from 1 (“strongly disagree”) to 4 (“strongly agree”). The MASS has demonstrated acceptable to excellent overall internal and test–retest reliability (*α* = 0.91, *r* = .88) as well as its four factors—counselor qualities (*α* = .91, *r* = .82), meeting needs (*α* = .72, *r* = .83), effectiveness (*α* = .85, *r* = .83), and counselor conflict (*α* = .74, *r* = .79) (Garland et al., 2000). Convergent validity with the Child Satisfaction Questionnaire-8 (CSQ-8) was high at *r* = .80, *p* < .001, divergent validity was found via a factor analysis where CSQ-8 items only loaded on the “effectiveness” subscale, and predictive validity was demonstrated as adolescents with unplanned discharges had lower satisfaction (Garland et al., 2000).

#### Therapeutic Alliance

It was collected using the Therapeutic Alliance Scale for Adolescents (TASA) and the Therapeutic Alliance Scale for Clinicians (TASC) at a 2-month follow-up (Faw et al., 2005). Both scales have 12 Likert scale items on a 6-point scale from 1 (“not at all true”) to 6 (“very true”) with two subscales: emotional bond and task collaboration. The adolescent and clinician TASA have good internal reliability (*α* = .85 and *α* = .84, respectively). Total score on therapist and client versions have been correlated at *r* = .33, *p* < .05 (Shirk et al., 2008), and the TASC was significantly correlated with observational coding of therapeutic alliance (*r* = .53, *p* < .02).

#### Student-Identified Top Problems

At baseline, the Youth Top Problem Assessment (YTPA; Weisz et al., 2011) asks youths to provide an open-ended response to “The three most important problems for which you need help.” At baseline and follow-up, the three problems are rated on a scale of 0 (“Not serious at all”) to 10 (“Very serious problem”). YTPA correlates with established symptom and functioning measures but with greater sensitivity to change, as well as good test–retest reliability (*r* = .69 to .91 for internalizing and externalizing scores; all *p* < .01). Most (79%) of youth-identified top problems were found to match caregiver-reported top problems; correlations between YTPA and the Youth Self Report (YSR) were found to be *r* = .33 and .37 for internalizing and externalizing (Weisz et al., 2011).

#### Mental Health Symptoms and Functioning

Mental health outcomes were measured using the Patient Health Questionnaire (PHQ-9; Richardson et al., 2010), the Generalized Anxiety Disorder Scale (GAD-7; Spitzer et al., 2006), Columbia Impairment Scale (CIS; Singer et al., 2011), and Brief Problem Checklist (BPC; Chorpita et al., 2010) at baseline, 2-, and 6-month follow-up. The PHQ-9 asks about the presence of nine criteria for depression disorder. Response options are on a 4-point scale from 0 (“not at all”) to 3 (“nearly every day"). Validity tests for the PHQ-9 have shown good sensitivity (.90) and specificity (.79) for detecting criteria for major depression on the Diagnostic Interview Schedule-IV (Richardson et al., 2010). Reliability scores in studies with adults have been found to be high (*ICC* = .88, *r* = .94; Zuithoff et al., 2010). As per cutoffs provided by the developers, a PHQ score ≥ 8.8 indicated moderate to severe depression.

The GAD-7 is a brief scale that queries about anxiety symptoms. Response options are on a 4-point scale from 0 (“not at all”) to 3 (“nearly every day"). Interrater reliability is good, with self-report scales correlated with clinician-administered versions (*r* = .83). Internal consistency has been high (Cronbach α as been high (Cronbach scales correl was good (ICC = .83) (Spitzer et al., 2006). The GAD-7 had high correlations with the Beck Anxiety Inventory (*r* = .72). The GAD-7 also had good sensitivity (.89) and specificity (.82) when tested against a full diagnostic interview. As per cutoffs provided by the developers, moderate to severe anxiety was indicated by a GAD-7 with a mean score of ≥ 10.

The CIS is a 13-item measure of adaptive functioning. Response options are on a 5-point scale from 0 (“no problem”) to 4 (“very big problem”). The CIS had good internal reliability (*α* = .80–.89) and demonstrated moderate construct validity with structured DMS diagnoses (kappa = .48; Singer et al., 2011). An impairment threshold of CIS with a mean score of ≥ 16 was used (Olfson, Druss, & Marcus, 2015).

The Brief Problem Checklist (BPC) is a youth-reported, 12-item questionnaire adapted from items on the Youth Self-Report (YSR; Achenbach & Rescorla, 2001). Response options are on a 3-point scale, from 0 (“not true”) to 2 (“very true”). Results are summed into total, internalizing, and externalizing subscales, with good internal consistency and 9-day test–retest reliability for each (*α* = .76, .72, .70; *r* = .79, .76, .72 respectively). Validity tests found correlations with the YSR above *r* = .60 and correlations with the caregiver-reported CBCL above *r* = .50 as well as significant associations with diagnoses obtained from a structured diagnostic interview (Chorpita et al., 2010).

#### Treatment Outcome

At the end of the third year of the study, SMHPs in both groups reported on the treatment outcome and triage decision for the five students with whom treatment was most recently completed. SMHPs reported whether, after four sessions, treatment was concluded or continued. For students who completed service, SMHPs reported on the treatment outcome from a list of five options: no further services planned, developed a monitoring plan, referred to other school services, referred to more intensive MH services, and referred to both school services and more intensive MH services.

#### BRISC Fidelity

The BRISC External Coding Tool (BECT) was developed for the study and includes 24 items derived from the 4 to 8 treatment components of each of the four BRISC sessions (e.g., “conducted stress rating”). Items are scored dichotomously (0 = criteria not met; 1 = met). For each BRISC clinician, the two BRISC trainer-consultants listened to audiotapes of all four sessions for at least the first two student clients each clinician enrolled and scored each session for fidelity criteria, overall quality, and student engagement on a 1–5 scale (i.e., 1 = low engagement/quality, 5 = high engagement/quality). Trainers double coded 22 recordings across all sessions (210 total items), achieving a single-measure absolute consistency of *ICC*_(2,1)_ = .916 (Koo & Li, 2016), indicating excellent reliability.

BRISC fidelity was also assessed by coding randomly selected sessions using the Therapy Process Observation Coding System (TPOCS; Garland et al., 2010). Three trained coders rated clinician use of 47 different types of intervention strategies on a 7-point scale from 0 (“not at all”) to 6 (“extensively”). All 47 TPOCS strategies were assigned by the BRISC developers to one of three codes: *consistent*, *divergent*, or *neither consistent with nor divergent* from BRISC (Bruns et al., 2019). For example, BRISC-convergent strategies included behavioral activation and skill building, BRISC-divergent strategies included advice and play therapy, and BRISC-neutral strategies included encouraging affect and functional analysis. To examine interrater reliability, 59 audio recordings were coded by all coders and considered adequate with a single-measure absolute consistency *ICC*_(2,1)_ of .671.

#### BRISC Feasibility and Acceptability

SMHPs were administered an exit interview after each school year that incorporated the Acceptability, Likely Effectiveness, Feasibility, and Appropriateness Questionnaire (ALFA-Q; Cook & Lyon, 2013), a 15-item measure that assesses the social validity (importance, acceptability, feasibility, likely effectiveness) of the intervention via numeric ratings on a five-point (0–4) Likert Scale. The ALFA-Q was developed by the research team based on the Treatment Acceptability Rating Form-Revised and Intervention Rating Profile-15, which have shown good reliability and validity (Martens et al., 1985; Reimer et al., 1991).

### Procedures

#### Randomization

To ensure condition comparability, we used stratified sampling. Within each state, a nearest neighbor matching algorithm matched schools based on characteristics of practitioners (e.g., degree, years’ experience, score on Evidence-Based Practices Attitudes Scale (Aarons, 2004)) and school (district, enrollment, % non-White, % standardized test scores, attendance). Schools were the randomization unit; a random number generator assigned one school from each match to BRISC or SAU. The analyst (MP) generated the random allocation and intervention assignment. Randomization successfully achieved balance, with no significant differences (at *p* < .1) for any of the above variables.

#### Recruitment and Enrollment

**Fig. 1:**
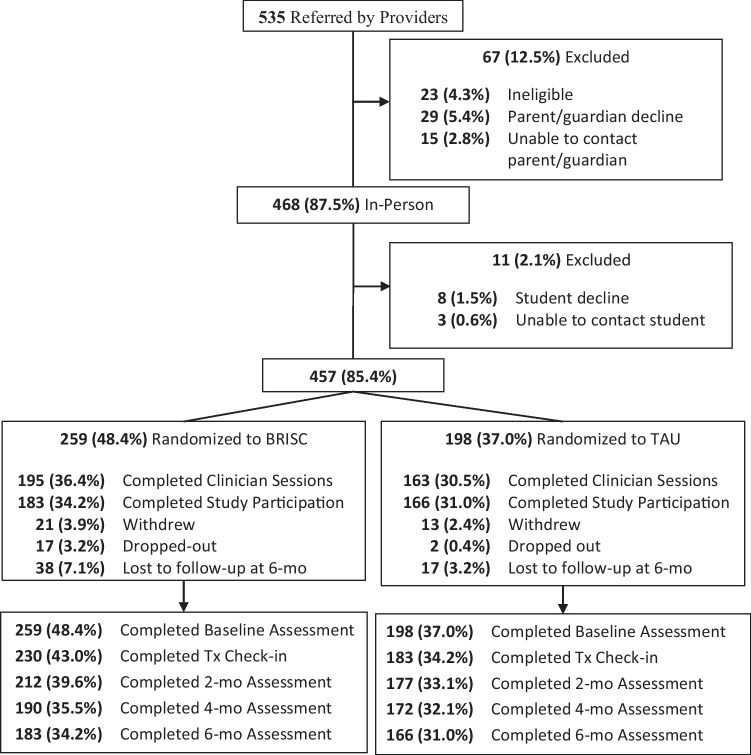
CONSORT diagram for student recruitment and study participation by condition (BRISC and SAU) in the BRISC study

Students were recruited and enrolled from October 2016 to November 2018. Figure 1 presents a CONSORT flowchart for the study (a CONSORT checklist is available as online resource 1). Participating SMHPs referred 535 high school students seeking/referred to SMH services; after 78 students (14.6%) were excluded due to ineligibility or inability to obtain consent, a total of 457 students attending 49 participating schools were enrolled in the study. Students either received BRISC (*N* = 259) or SAU (*N* = 198) based on the study arm of their school. The mean number of students enrolled by the school was 10.5 (*SD* = 9.5), with a range of 1–34. The mean number of students enrolled by SMHP was 8.5 (*SD* = 6.5), with a range from 1 to 29.

#### Data Collection

Study procedures were approved by the Institutional Review Board at the University of Washington. Informed consent was obtained from students, caregivers, and SMHPs. Students completed measures at baseline, 2, and 6 months. Briefer surveys (YTPA only) were also conducted with students at 1 and 4 months. At the end of each treatment episode, all SMHPs completed the TASC; BRISC SMHPs also completed the feasibility self-report measures. All SMHPs completed implementation measures (including the ALFA-Q) and qualitative surveys at the end of each year.

#### BRISC Intervention

The core BRISC strategy is implemented in four sessions. In session 1, the SMHP engages the student, assesses current functioning using brief standardized assessment measures, and identifies “top problems” (Weisz et al., 2011). The student is asked to informally monitor a behavior related to one of their top problems (e.g., time spent with friends, days feeling “blue,” number of disputes with parents). In session 2, the SMHP introduces a structured problem-solving framework, and the student chooses a “top problem” to address. Using a structured approach, the student outlines a concrete goal, brainstorms possible steps toward this goal, identifies a specific step, and troubleshoots barriers (D'Zurilla & Nezu, 2010). In session 3, a set of evidence-based, skill-based elements (communication skills, stress and mood management, realistic thinking) are taught, if needed, to help modify the step tried or to identify and support the student’s next step(s). In session 4, the student and SMHP review progress and identify a “post-BRISC pathway”: (1) end treatment/problem resolved, (2) supportive monitoring by the SMHP or other school staff, (3) continued treatment from the SMHP, or (4) referral to more specialized or intensive services. SMHPs are also encouraged to refer students to supportive school-based services (e.g., tutoring, special education), if indicated.

BRISC-assigned SMHPs attended a 1.5-day in-person training by two Ph.D.-level clinical psychologists that incorporated strategies (e.g., modeling, role play) found to facilitate uptake of new skills (Rakovshik & McManus, 2010). Although therapists in both conditions were already deployed full time by their agencies into the school setting, the training also reviewed how to manage the unique challenges of SBMH work as it applies to the BRISC framework (e.g., adjusting to school schedules, triaging to school and community supports). SMHPs received bi-weekly phone consultation/coaching from the trainers that included a review of adherence checklists completed by the clinician following each of their sessions, case presentations, and review of and feedback on their BRISC implementation.

### Services as Usual

Students in SAU-assigned schools received individual counseling/therapy as usual from SMHPs. Based on previous research, the primary orientation of SMHPs in the SAU condition is supportive psychotherapy and crisis response (Holmes et al., 2021; Lyon et al., 2015). SMHPs in SAU-assigned schools likely used a diverse array of treatment strategies including some directive, skill-building techniques common in EBPs, but at an overall lower rate and intensity than in BRISC or other EBPs.

## Data Analysis

Overall, attrition and differential attrition were calculated to examine potential selection bias. Mixed effects modeling with random effects at the clinician and client level were used for statistical testing of between-group differences. If models failed to converge, resulted in Hessian errors, or had no significant variance at these levels, these random effects were removed. Standard model building procedures were used (Raudenbush & Bryk, 2002; Singer & Willett, 2003). The sole predictor for cross-sectional analyses (e.g., TASA, MASS) was condition (BRISC versus SAU). Predictors for longitudinal outcomes (e.g., GAD-7, PHQ) included time, condition, and a time x condition interaction term. If data screening of longitudinal time trends indicated potential curvilinearity, we included a quadratic term and condition interaction. Models were fit using restricted maximum likelihood estimation. Dichotomous outcomes (e.g., any services received) were modeled using binomial distribution with logit link function. All analyses were intention-to-treat, and no members of originally assigned groups changed conditions.

## Results

### Condition Characteristics

As shown in Table 1, there were no significant group differences at baseline by study condition for any of the student variables except for race; further examination indicated this was solely due to more Black students in BRISC (34.7% vs. 19.8%, *p* < .05). Overall, 87.3% of study-enrolled students scored above clinical cutoffs for one or more of the symptom/functioning measures (GAD-7, PHQ-9, CIS). A total of 413 students (85.1%) initially enrolled in the study were successfully assessed at a 2-month follow-up, and 395 (76.3%) were retained and assessed at 6 months. Study attrition was significantly higher for BRISC than for SAU at 2 month (18.2% vs. 10.6%, *p* < .05) and 6-month follow-up (29.4% vs. 16.7%; *p* < .01).

**Table 1:** Student demographics by condition

	BRISC (*n* = 259)		SAU (*n* = 198)		Total (*n* = 457)		*p*
	*n*	%	*n*	%	*n*	%	
Gender							0.302
Male	91	35.1	58	29.3	149	32.6	
Female	165	63.7	140	70.7	305	66.7	
Endorsed another gender	3	1.2	0	0	3	0.7	
Race							0.037
American Indian/Alaskan Native	2	0.8	4	2.0	6	1.3	
Asian	11	4.2	10	5.1	21	4.6	
Black/African American^b^	90	34.7	39	19.8	129	28.3	
Native Hawaiian/Pacific Islander	2	0.8	4	2.0	6	1.3	
White/Caucasian	88	34.0	42.1	83	171	37.5	
Latinx as race only^a^	29	11.2	29	14.7	58	12.7	
Multiracial	33	12.7	24	12.2	57	12.5	
Other^a^	4	1.5	4	2.0	8	1.8	
Ethnicity–Latinx	49	18.9	49	24.7	98	21.4	0.225
Grade							0.776
9th Grade	85	32.8	55	27.8	140	30.6	
10th Grade	59	22.8	55	27.8	114	24.9	
11th Grade	65	25.1	54	27.3	119	26.0	
12th Grade	50	19.3	34	17.2	84	18.4	
Free/Reduced Lunch Eligible	161	62.2	117	59.1	278	60.8	0.365
Symptom cutoffs							
PHQ-9 above cutoff	145	56.2	125	63.1	270	59.2	0.136
GAD-7 above cutoff	134	51.9	106	53.5	240	52.6	0.735
CIS above cutoff	137	53.1	105	53.0	242	53.1	0.988
Any symptom measure above cutoff	230	89.1	168	84.8	398	87.3	0.172
Symptom levels							
PHQ-9 (M, SD)	10.2 ± 6.1		11.1 ± 6.3		10.6 ± 6.2		0.131
GAD-7 (M, SD)	10.1 ± 5.3		10.2 ± 5.6		10.1 ± 5.4		0.817
CIS (M, SD)	16.8 ± 9.1		16.7 ± 9.4		16.7 ± 9.2		0.890
Age (M, SD)	16.3 ± 1.3		16.2 ± 1.1		16.3 ± 1.2		0.477

*PHQ-9* Patient Health Questionnaire, *GAD-7* Generalized Anxiety Disorder-7, *CIS* Columbia Impairment Scale

^a^Youth who endorsed “Latino as race only” selected Latino ethnicity and either did not select a race or selected “other” and endorsed Latinx or a Latinx country

^b^Adjusted standardized residual > 1.96 or <  − 1.96; this row is significantly larger or smaller than expected

### BRISC Fidelity

Fidelity ratings via the BECT found excellent adherence for sessions 1, 2, and 4, at 94.2%, 90.8%, and 90.1% of items in each session, respectively. Adherence was acceptable but lower for session 3 with 77.4% of items meeting criteria. Session 3 focused on continued problem solving and skill-based elements of mental health care are taught to facilitate step completion. The lowest fidelity items for session 3 were “create a game plan for the coming week” (74.6% of sessions) and “obtained stress rating associated with identified problem” (89.8% of sessions). Items regarding fidelity for problem-solving tasks were high (98.3% of the sessions). Session quality ranged from 3.0 (session 3) to 3.7 (session 1) on the 5-point scale, and engagement ranged from 3.9 (session 3) to 4.2 (session 1) on the 5-point scale.

Mixed-effect modeling of TPOCS scores confirmed treatment fidelity. Sessions from the BRISC condition featured more elements *consistent* with BRISC (BRISC mean = 2.10, SAU mean = 1.66, *p* < .001) and fewer elements *divergent* from BRISC training (BRISC Mean = 1.06, SAU Mean = 1.17, *p* < .001). No significant differences were found for elements *neither consistent with nor divergent from* BRISC (BRISC mean = 1.58, SAU mean = 1.55, *p* = .448). These analyses provide evidence for internal validity by verifying treatment condition adherence via masked observations, proving that the services received in the BRISC condition differed from treatment as usual.

### BRISC Feasibility and Acceptability

Results of anonymous exit surveys indicated that SMHPs perceived BRISC to be feasible and acceptable. Mean ratings across all items on the ALFA-Q were 3.05 (*SD* = .15) on a 0–4 scale. ALFA-Q item scores ranged from a low of 2.48 (“How compatible do you find BRISC to be with the practical realities and resources of working in the school setting?”) to a high of 3.51 (“How useful was the training, information, and support to aiding your implementation of BRISC?”). Mean satisfaction with BRISC was 3.09 (*SD* = 0.66); mean “compatibility with the school’s mission” was 3.28 (SD = 0.81); and mean rating of “the extent to which BRISC is likely to improve students’ social, emotional, and academic success” was 3.08 (*SD* = .76). A full report on BRISC feasibility and acceptability is available as online resource 2.

### Service Processes

Therapeutic alliance as evaluated by total scores of the TASA and TASC and youth satisfaction as evaluated by the MASS total score and its four subscales found no significant differences, with one exception: Students in SAU rated their SMHP significantly higher than students in BRISC on the Counselor Qualities subscale of the MASS (*M* = 3.57 vs. *M* = 3.46, *p* = .038). 

### Services Received

#### Student Report

**Fig. 2:**
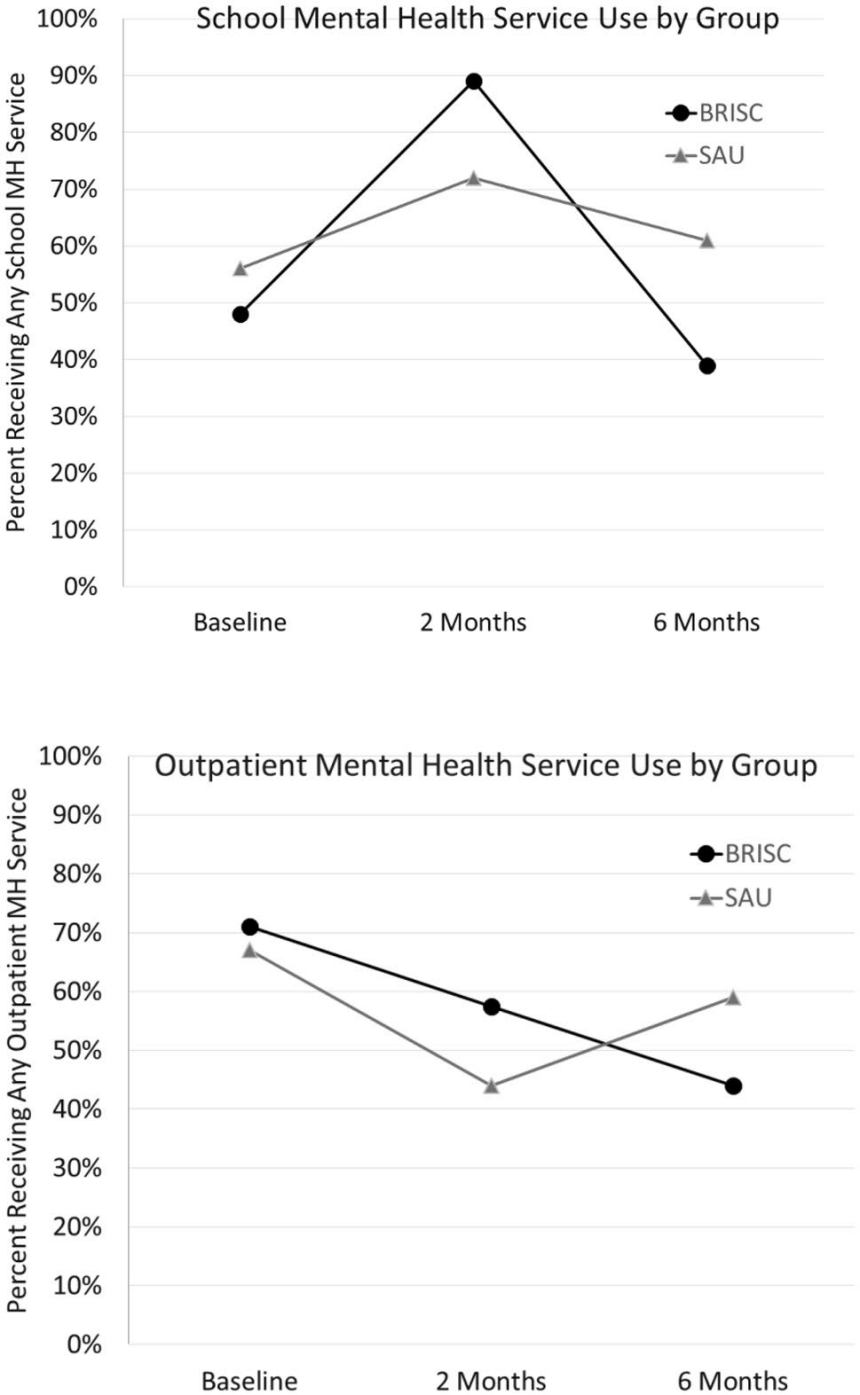
Rate of school and outpatient mental health service receipt over time for BRISC and services as usual (SAU)

Results of analyses of longitudinal service receipt as assessed via the SACA are shown in Table 2 and Fig. 2 (model results with confidence intervals are available as online resource 3). There were no differences at baseline between conditions on the proportion of students receiving school-based mental health services. From baseline to 2 months, the proportion of students receiving SMH services, and the number of services received, increased for both conditions. From 2 to 6 months, the proportion of students receiving SMH and the number of SMH services decreased more for the BRISC condition. At 6 months, there were no significant differences between the groups on the proportion of students receiving SMH services, but the number of SMH services received was significantly less for BRISC. This demonstrates that BRISC was associated with increased SMH services between baseline to 2 months and decreased SMH services by 6 months. The proportion of BRISC students who used outpatient MH services significantly decreased across all time points, while the proportion of students in SAU receiving outpatient services decreased from baseline to 2 months and then increased. At 6 months, significantly fewer BRISC students received outpatient services.

**Table 2:** Coefficients from mixed-effect models predicting any services received and number of services received^a^

**Outcome**	**6-month intercept**	**BRISC**^b^	**Time**^b^	**BRISCx x time**^b^	**Time**^**2**^	**BRISC x time**^**2**^
Any services^a^						
School services	.498	−.316	−.626***	−1.491***	−.167***	−.375***
Outpatient services	.391	−.652*	.746***	−.983***	.209***	−.193***
Inpatient services	−3.483***	−.691	−.047	.623	−.035	.283
All services	1.402***	−.509	−.098	−1.510***	.003	−.349***
Number of services^a^						
School services	.781***	−.176*	−.112*	−.341***	−.030*	−.080***
Outpatient services	.824***	−.236*	.287***	−.328**	.092***	−.058**
All services	1.654***	−.427**	.153	−.622***	.055**	−.121***

^*^p ≤ .05; ^**^p ≤ .01; ^***^p ≤ .001

^a^Full model results available as online resource 3. “Number of services” models applied linear mixed modeling. Inpatient services were excluded from analyses due to the small number of students who received more than one inpatient service. “Any services” models applied generalized mixed models with a logit link

^b^Time is centered at 6-month follow-up, therefore significant differences in BRISC indicate a difference between the groups at follow-up

#### Clinician Report

Clinician report of client status after 4 sessions was limited to the 9 BRISC and 13 SAU SMHPs still participating in the study in the final year of the study (*N*=45 and *N*=65 students, respectively). As shown in Table 3, there was a significant difference after 4 sessions (*χ*^2^ = 18.9, *p* = .004), with adjusted standardized residuals indicating students in the BRISC condition were more likely than SAU to have concluded treatment with no further services planned (37.8% vs. 12.3%, *RR* = 3.07, *RD* = 0.25) and to be referred to outside MH services (15.6% vs 3.1%, *RR* = 5.06, *RD* = 0.12). SAU students were more likely to continue school-based treatment with no additional services planned (61.5% vs. 35.6%, *RR* = 0.58, *RD* =  −0.26).

**Table 3:** Clinician-reported service dispositions for youth in the BRISC condition versus services as usual (SAU)

	BRISC (*n* = 45)		SAU (*n* = 65)		Total sample (*n* = 110)		Relative risk	Risk difference
	*n*	%	*n*	%	*n*	%		
**Concluded treatment**								
No further services/follow-up planned^a^	17	37.8	8	12.3	25	22.7	3.07	0.25
Developed a monitoring plan	3	6.7	9	13.8	12	10.9	0.49	−0.07
Referred to other school services	2	4.4	4	6.2	6	5.5	0.72	−0.02
Referred to more intensive MH^a^	7	15.6	2	3.1	9	8.2	5.06	0.12
Referred to other school services and more intensive MH services	0	0.0	1	1.5	1	0.9	–	−0.02
**Continued school-based treatment**								
No additional services planned^a^	16	35.6	40	61.5	56	50.9	0.58	−0.26
Refer to addl school services	0	0.0	1	1.5	1	0.9	–	−0.02
Multiple services selected	1	2.2	4	6.2	5	4.5	0.36	−0.04

Chi-square = 18.9, *p* = .004

^a^Adjusted standardized residual for the cell is > 1.96 or <  − 1.96, indicating this row is significantly larger or smaller than expected

### Student-Identified Top Problems

**Fig. 3:**
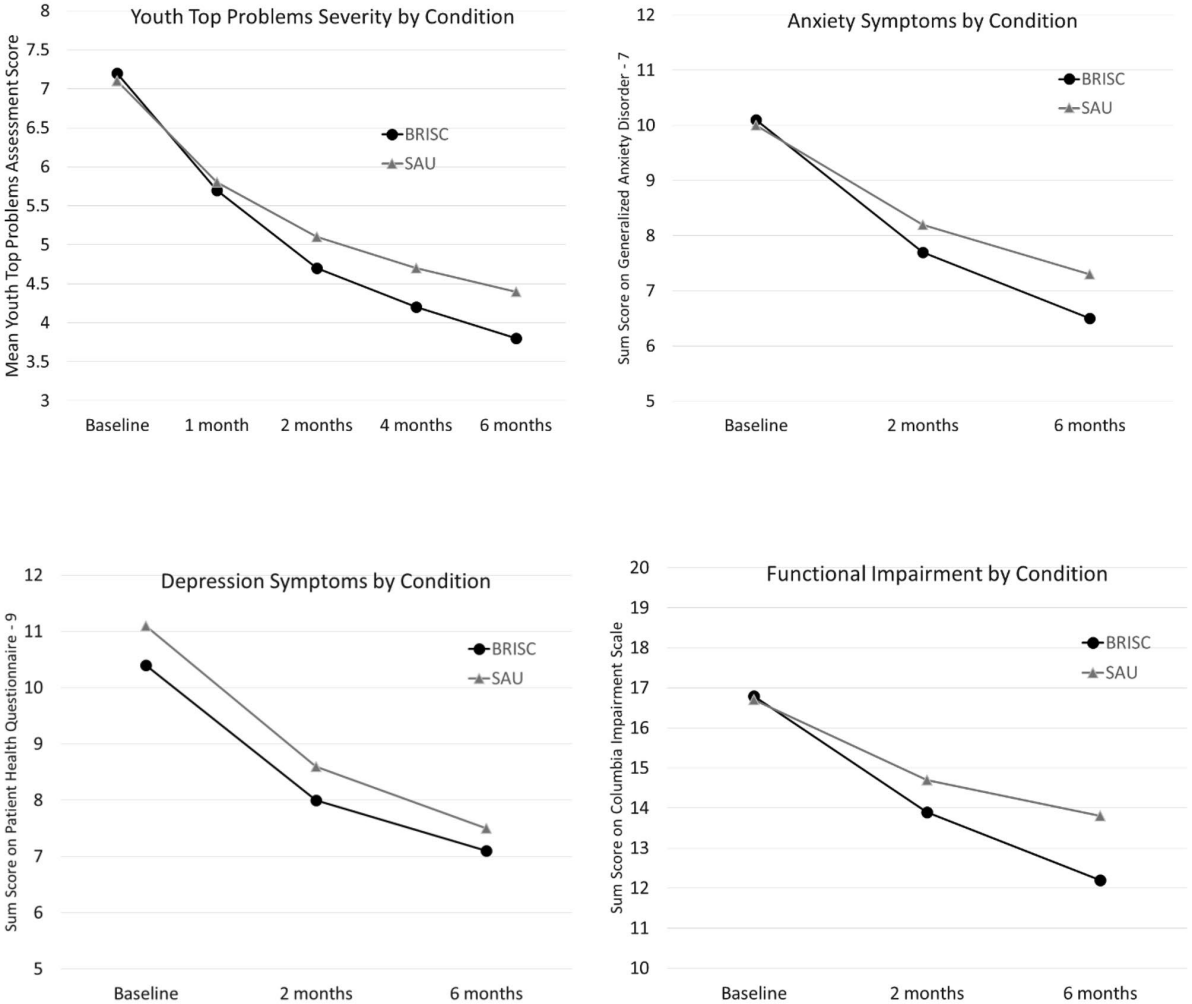
Mental health outcomes over time by condition

As shown in Fig. 3, both conditions rated their YTPA top problems as near-equivalent seriousness at baseline; however, BRISC students demonstrated greater improvement at both 2- and 6-month follow-up. As presented in Table 4 and online resource 4, results of individual mixed-effect models indicated both conditions significantly improved (Coeff =  −0.71, *p* < .01), but there was a significant condition x time effect; students in the BRISC group had a sharper reduction in seriousness (Coeff =  −0.16, *p* < .01) and had significantly lower scores at 6-month follow-up (Coeff =  −0.60, *p* < .01).

**Table 4:** Coefficients from individual mixed effects models predicting mental health outcomes for all youth and caregivers in the BRISC condition versus services as usual (SAU)^a^

**Outcome**	**6-month intercept**	**BRISC**^b^	**Time**^b^	**BRISC x time**^b^
Youth Top Problems Assessment	4.099^**^	−0.601^**^	−0.705^**^	−0.164^**^
Brief Problem Checklist (BPC)				
BPC externalizing	0.458^**^	−0.005	−0.018^*^	−0.014
BPC internalizing	0.728^**^	−0.112^*^	−0.041^**^	−0.017
BPC total score	0.592^**^	−0.058	−0.029^**^	−0.015
Generalized Anxiety Disorder	7.060^**^	−0.737	−0.770^**^	−0.152
Patient Health Questionnaire	7.470^**^	−0.515	−0.900^**^	0.086
Columbia Impairment Scale	13.432^**^	−1.248	−0.800^**^	−0.333

^*^p ≤ .05; ^**^p ≤ .01

^a^Full model results available as online resource 4

^b^Time is centered at 6-month follow-up; therefore, significant differences in BRISC indicate a difference between the groups at follow-up

### Mental Health Symptoms and Functioning

Table 4 summarizes longitudinal mixed-effect models for all students on youth symptoms and functioning (for full results, see online resource 4). Figure 3 presents a visual depiction of longitudinal trends by condition. Both conditions showed significant improvements on all scales. Students in BRISC demonstrated lower scores on the BPC Externalizing scale at 6 months, but did not show a significantly different rate of improvement, despite statistical equivalence at baseline. There were no significant differences between conditions for the BPC Internalizing, BPC Total Score, GAD-7, PHQ-9, or CIS.

## Discussion

This project evaluated the potential for improving the efficiency of SMH via a school-based assessment, brief intervention, and triage approach for students with socio-emotional concerns. BRISC provides a first-line intervention using consistent assessment to inform level and type of ongoing services needed in a prevention-oriented, multi-tiered delivery model. Research questions focused on feasibility, fidelity, student report of problems, and ability of BRISC to promote efficiency within SMH. We also measured a range of mental health outcomes over 6-month follow-up to evaluate whether this emphasis on efficiency compromised these outcomes compared to SMH “as usual.”

SMHPs who participated in the study were predominantly outpatient clinic providers deployed to the school setting. Their training and established practice were geared to traditional, longer term supportive therapy. The structured, measurement-based, and goal-oriented BRISC approach represented a different way of practicing aligned with data-informed, multi-tiered frameworks used by schools. Despite the learning curve required and deviation from typical practice, SMHPs rated BRISC as feasible, acceptable, and appropriate. Importantly, the majority of SMHPs rated BRISC as easy to learn, “compatible with the school mission,” and “likely to improve students’ social, emotional, and academic success.” SMHPs delivered BRISC with fidelity; however, session 3 fidelity was lower than for other sessions. This was likely due to the complexity introduced by the option to use evidence-based treatment elements as needed.

A second key finding was that SMHPs in the BRISC condition were significantly more likely (53% to 15%) to report that they had completed treatment after 4 sessions than SAU. BRISC-assigned SMHPs were also more likely to refer students to alternative and/or more intensive services. Students in the BRISC condition were also significantly more likely to report receipt of SMH at 2 months and less likely to report SMH services at 6 months. These results support the hypothesis that BRISC can promote SMH that is more efficient and better aligned with MTSS and public health frameworks.

Finally, we asked whether SMHPs’ use of a brief strategy to achieve greater efficiency would compromise mental health outcomes. To the contrary, students served via BRISC showed significantly greater improvement on their self-rated top mental health problems at 2 and 6 months. Symptom and functioning measures showed significant improvement, but slopes did not differ by condition. Students served by BRISC SMHPs were less likely to receive outpatient care at 6 months. This finding could indicate that BRISC more effectively addressed students’ “top problems” to an extent that reduced their need for longer-term mental health services. Or, it could be that students in the BRISC condition felt more confident in their school-based provider to help them in the future, reducing the need for clinic-based services. Such questions will need to be examined in more in-depth research with longer follow-up.

### Limitations

Although the current study used rigorous methods, attrition from follow-up data collection was not trivial (15% at 2 months and 22% at 6 months) and significantly higher for students assigned to BRISC. Debriefs with interviewers suggested that SAU students were easier to schedule for follow-up interviews due to their continued mental health treatment, whereas more BRISC students had completed treatment by 3–6 months, making follow-up data collection more difficult. It is possible those lost to follow-up were actually more likely to have improved, reducing the effects found for BRISC.

Second, SMHPs in the BRISC condition were explicitly trained to take stock of next steps after four sessions, meaning that the greater rate of treatment completion (53% to 15%) could be viewed as a demand characteristic in effect for just the BRISC condition. However, SMHPs in the BRISC condition were trained only to evaluate progress and triage as appropriate after four sessions, ending treatment only if progress data suggested it was warranted. As such, we do not believe this is an artifact of study design.

Third, because treatment logs maintained by SMHPs were found to have been inconsistently maintained, the core outcome of treatment pathway after 4 sessions was only available via SMHP report after the third year of the study, limiting this outcome to only 22 of the 53 enrolled SMHPs. Finally, despite the multiplicity of analyses, we did not adjust for familywise error. Doing so over dozens of tests would have resulted in an unacceptable Type II error rate and presented an incomplete picture of results.

### Implications for School-Based Prevention and Treatment

Results indicate that BRISC holds promise as a method for equipping SMHPs with a structured approach to meeting the needs of a greater number of students with an array of problems. Developing such targeted strategies is important given that there are a limited number of such “Tier 2” SMH strategies available in schools (Mitchell, Stormont, & Gage, 2011), with most addressing behavioral concerns such as disruption and off-task behavior. Youth with internalizing problems such as anxiety and depression are less likely to be identified and receive intervention (Splett et al., 2019).

It is important to note that the components of BRISC—structured problem solving, standardized assessment, stepped care, and measurement-based care—are all “common elements” of evidence-based mental health treatment. Although BRISC was designed to help SMHPs incorporate these strategies over a sequence of four defined sessions, incorporating any one of these strategies into SMH may promote efficiency and effectiveness. Future research may shed light on how best to equip a range of school-employed helpers with one or more of these elements to help them respond more proactively to a greater number of students. Ideally, future research will also evaluate contextual factors such as fidelity to MTSS principles and procedures such as universal screening. Such data could uncover the degree to which fidelity and outcomes of a strategy such as BRISC are related to district- and school-level factors and how to better integrate SMH strategies.

Finally, it is important to note that providing access to counseling is not, in and of itself, a solution to the rising rates of mental health concerns of youth. While treatment models such as BRISC may aim to serve as one element in a tiered continuum of data-driven, evidence-informed school strategies, they are also reactive and resource-intensive. They do not, as described by Herman et al. (2021), “alter the contextual factors in the broader social environment that contribute to, maintain, and exacerbate the problem in the first place.” Although BRISC shows promise, ideally, it would be one component of a comprehensive, multi-tiered system. Given the rising rates of youth mental health challenges, it is even more critical to invest in comprehensive frameworks such as MTSS that may be able to reduce the number of youths requiring targeted services.

## Supplementary Information

Below is the link to the electronic supplementary material.

Supplementary file1 (DOC 219 KB)

Supplementary file2 (DOCX 14 KB)

Supplementary file3 (DOCX 16 KB)

Supplementary file4 (DOCX 16 KB)
