# Supplementary material for: Effectiveness of a Brief Engagement, Problem-Solving, and Triage Strategy for High School Students: Results of a Randomized Study
Source: Prev Sci. 2023 Mar 17;24(4):701–14. doi: 10.1007/s11121-022-01463-4 (PMC10227122; doi:10.1007/s11121-022-01463-4)
Supplement: Supplementary file 2 — Supplementary file2 (DOCX 14 KB) [file 11121_2022_1463_MOESM2_ESM.docx]

**Online resource 2.** Detailed Report of Acceptability and Feasibility Ratings from School Practitioners (N=53) Trained on the Brief Intervention for School Clinicians (BRISC)

|  |  | | |
| --- | --- | --- | --- |
| **Item** |  | | |
|  | **Mean** | **SD** | **Range** |
| 1. To what extent are you satisfied with the content of BRISC? | 3.09 | 0.658 | 3-4 |
| 2. To what extent do you believe your fellow school clinicians would be satisfied with BRISC? | 2.79 | 0.840 | 1-4 |
| 3. How credible did you find BRISC? | 3.28 | 0.601 | 2-4 |
| 4. How credible do you believe fellow school clinicians will find BRISC? | 2.92 | 0.781 | 1-4 |
| 5. How well organized and delivered did you find the content of BRISC? | 3.36 | 0.736 | 2-4 |
| 6. How well organized do you believe fellow school clinicians would find the information and support provided to implement the intervention? | 3.13 | 0.810 | 2-4 |
| 7. How comfortable are you with using BRISC? | 2.98 | 0.930 | 2-4 |
| 8. How comfortable do you believe fellow school clinicians will be with using BRISC? | 2.77 | 0.933 | 1-4 |
| 9. How compatible do you find BRISC to be with the practical realities and resources of working with students in the school setting? | 2.64 | 1.002 | 2-4 |
| 10. How compatible do you believe fellow school clinicians will find BRISC to be with the practical realities and resources of the school setting? | 2.48 | 1.000 | 1-4 |
| 11. Overall, how feasible do you believe BRISC is for use by clinicians and counselors working in school settings? | 2.85 | 0.907 | 2-4 |
| 12. How compatible is BRISC with the school’s mission and expectations to support the academic success of students? | 3.28 | 0.818 | 1-4 |
| 13. How relevant is BRISC to the implementation of practices that focus on removing social, emotional, and behavioral barriers to academic success? | 3.08 | 0.917 | 2-4 |
| 14. Overall, how relevant do you believe BRISC is to improving school-based supports and services for students who are at-risk? | 3.02 | 0.909 | 2-4 |
| 15. How useful was the training, information, and support to aiding your implementation of BRISC? | 3.51 | 0.697 | 3-4 |
| 16. To what extent do you believe BRISC is likely to improve students’ social, emotional, and academic success? | 3.08 | 0.756 | 2-4 |
| 17. Overall, to what extent will BRISC as a whole effectively enable school clinicians to use effective practices that target at-risk students’ social, emotional, and academic success? | 2.94 | 0.842 | 1-4 |
