# Supplementary material for: Effectiveness of a Brief Engagement, Problem-Solving, and Triage Strategy for High School Students: Results of a Randomized Study
Source: Prev Sci. 2023 Mar 17;24(4):701–14. doi: 10.1007/s11121-022-01463-4 (PMC10227122; doi:10.1007/s11121-022-01463-4)
Supplement: Supplementary file 3 — Supplementary file3 (DOCX 16 KB) [file 11121_2022_1463_MOESM3_ESM.docx]

**Online resource 3. Detailed model results for longitudinal service receipt as assessed via the Services Assessment for Children and Adolescents (SACA)**

|  | **6-month Intercept** | | **BRISC^b^** | | **Time^b^** | | **BRISC**  **x Time^b^** | | **Time^2^** | | **BRISC x Time^2^** | |
| --- | --- | --- | --- | --- | --- | --- | --- | --- | --- | --- | --- | --- |
| **Outcome** | **Coeff** | **95% CI** | **Coeff** | **95% CI** | **Coeff** | **95% CI** | **Coeff** | **95% CI** | **Coeff** | **95% CI** | **Coeff** | **95% CI** |
| Any Services^a^ |  |  |  |  |  |  |  |  |  |  |  |  |
| School Services | .498 | -.014, 1.011 | -.316 | -.900, .268 | -.626*** | -.932, -.320 | -1.491*** | -2.184, -.798 | -.167*** | -.256, -.077 | -.375*** | -.570, -.179 |
| Outpatient Services | .391 | -.039, .822 | -.652* | -1.236, -.068 | .746*** | .423, 1.070 | -.983*** | -1.394, -.571 | .209*** | .133, .286 | -.193*** | -.291, -.096 |
| Inpatient Services | -3.483*** | -4.260, -2.707 | -.691 | -2.118, .736 | -.047 | -.780, .685 | .623 | -1.297, 2.543 | -.035 | -.234, .164 | .283 | -.167, .733 |
| All Services | 1.402*** | .854, 1.949 | -.509 | -1.164, .146 | -.098 | -.455, .260 | -1.510*** | -2.158, -.862 | .003 | -.084, .090 | -.349*** | -.504, -.195 |
| Number of Services^a^ | | |  |  |  |  |  |  |  |  |  |  |
| School Services | .781*** | .668, .894 | -.176* | -.332,-.021 | -.112* | -.221, -.002 | -.341*** | -.490, -.190 | -.030* | -.056, -.005 | -.080*** | -.114, -.045 |
| Outpatient Services | .824*** | .671, .977 | -.236* | -.446, -.025 | .287*** | .147, .425 | -.328** | -.519, -.137 | .092*** | .059, .124 | -.058** | -.102, -.014 |
| All Services | 1.654*** | 1.440, 1.872 | -.427** | -.726, -.127 | .153 | -.039, .345 | -.622*** | -.885, -.358 | .055** | .011, .099 | -.121*** | -.181, -.060 |
