# Supplementary material for: Effectiveness of a Brief Engagement, Problem-Solving, and Triage Strategy for High School Students: Results of a Randomized Study
Source: Prev Sci. 2023 Mar 17;24(4):701–14. doi: 10.1007/s11121-022-01463-4 (PMC10227122; doi:10.1007/s11121-022-01463-4)
Supplement: Supplementary file 4 — Supplementary file4 (DOCX 16 KB) [file 11121_2022_1463_MOESM4_ESM.docx]

**Online Resource 4.** Detailed individual mixed effects model results for longitudinal assessment of student outcomes.

|  | **6-month Intercept** | | **BRISC^b^** | | **Time^b^** | | **BRISC x Time^b^** | |
| --- | --- | --- | --- | --- | --- | --- | --- | --- |
| **Outcome** | **Coeff** | **95% CI** | **Coeff** | **95% CI** | **Coeff** | **95% CI** | **Coeff** | **95% CI** |
| Youth Top Problems Assessment | 4.099^**^ | 3.815, 4.384 | -0.601^**^ | -0.990, -0.213 | -0.705^**^ | -0.801, -0.213 | -0.164^**^ | -0.294, -0.034 |
| Brief Problem Checklist (BPC) |  |  |  |  |  |  |  |  |
| BPC Externalizing | 0.458^**^ | 0.403, 0.512 | -0.005 | -0.079, 0.070 | -0.018^*^ | -0.034, -0.002 | -0.014 | -0.036, 0.008 |
| BPC Internalizing | 0.728^**^ | 0.649, 0.806 | -0.112^*^ | -0.218, -0.005 | -0.041^**^ | -0.063, -0.018 | -0.017 | -0.047, 0.013 |
| BPC Total Score | 0.592^**^ | 0.538, 0.647 | -0.058 | -0.132, 0.017 | -0.029^**^ | -0.045, -0.013 | -0.015 | -0.037, 0.006 |
| Generalized Anxiety Disorder | 7.060^**^ | 6.260, 7.856 | -0.737 | -1.822, 0.349 | -0.770^**^ | -0.984, -0.557 | -0.152 | -0.443, 0.138 |
| Patient Health Questionnaire | 7.470^**^ | 6.573, 8.368 | -0.515 | -1.736, 0.707 | -0.900^**^ | -1.131, -0.670 | 0.086 | -0.227, 0.399 |
| Columbia Impairment Scale | 13.432^**^ | 12.093, 14.772 | -1.248 | -3.070, 0.575 | -0.800^**^ | -1.142, -0.459 | -0.333 | -0.787, 0.132 |
